# Acupotomy for patients with carpal tunnel syndrome: A systematic review protocol

**DOI:** 10.1097/MD.0000000000018336

**Published:** 2019-12-20

**Authors:** Qiaoyin Zhou, Yifeng Shen, Xiaojie Sun, Zuyun Qiu, Yan Jia, Shiliang Li

**Affiliations:** a College of Traditional Chinese Medicine, Fujian University of Traditional Chinese Medicine, Fuzhou, Fujian; b Department of acupuncture-moxibustion, China Japan Friendship hospital; c Hospital of Chengdu University of Traditional Chinese Medicine, Chengdu, Sichuan, China.

**Keywords:** acupotomy, carpal tunnel syndrome, protocol, systematic review

## Abstract

**Background:**

This systematic review program is designed to provide an assessment of the effectiveness and safety of needle-knife therapy for the treatment of carpal tunnel syndrome (CTS).

**Methods:**

A cumulative search till October 2018 will be conducted in the following 8 databases: PubMed, EMBASE, Cochrane Controlled Trials Register, MEDLINE, China National Knowledge Infrastructure, China Biomedical Literature Database, VIP Database, and Wanfang Database, with no language or Publication status restrictions. All randomized controlled trials (RCT) for carpal tunnel syndrome will be considered eligible. The primary outcomes will include changes in the Boston carpal tunnel questionnaire (BCTQ) and visual analogue score (VAS), as well as safety and adverse events. Study inclusion, data extraction, and quality assessment will be done independently by 2 reviewers. If no substantial heterogeneity is detected, a meta-analysis will be performed. Continuous results will be expressed as mean differences or standard average differences, while binary data will be expressed as relative risks. The deviation risk and data synthesis will be assessed using the Review Manager software.

**Results:**

This study will provide a high-quality synthesis of BCTQ and VAS to assess the effectiveness and safety of acupotomy for carpal tunnel syndrome patients.

**Conclusion:**

This systematic review will provide evidence to judge whether acupotomy is an effective intervention for patients with carpal tunnel syndrome.

**PROSPERO registration number:**

CRD42018108787.

## Abstract 2

Supplemental Digital Content is available in the text

## 1 Introduction

Carpal tunnel syndrome (CTS), also known as late-onset median nerve palsy, is usually caused by compression of the median nerve in the carpal tunnel. The main symptoms are pain in the thumb, index finger and middle finger, numbness, and paresthesia, and dysfunction of a substantial area of the hand, innervated by the medial nerve. Carpal tunnel syndrome has gradually become a common peripheral nerve compression syndrome. Demographically, 80% of patients are over 40 years old, women are significantly more commonly affected than males, and approximately 50% of patients have bilateral involvement.^[1]^ This makes it a serious public health problem, and CTS has been receiving widespread clinical attention because of this high incidence and significant disability.^[2]^

According to the 2016 American Academy of Orthopaedic Surgeons (AAOS) guidelines for the management of carpal tunnel syndrome, current treatment options for CTS are diverse and generally divided into conservative and surgical therapies.^[3]^ Conservative options include fixed wrist brace, local corticosteroid injection, and acupuncture. The AAOS guidelines state that conservative treatment is the treatment of choice for patients with mild to moderate CTS, and surgery is only required when it fails.^[4]^ Although CTS has a variety of therapeutic interventions, their therapeutic effect is not satisfactory.^[5–8]^

Acupotomy therapy is a modern treatment technology developed with advances in medical technology. Acupotomy is a miniature surgical instrument consisting of a handle, a needle, and a blade.^[9]^ It can be used to cut and separate abnormalities, scars, and contractures with micro-trauma.^[10]^ Acupotomy treatment of CTS has its unique advantages and has been widely used in the treatment of CTS in Chinese medicine, orthopedics, and pain clinics, with satisfactory results.^[11–14]^ Some trials^[11,15,16]^ have reported that acupotomy can effectively release the transverse ligament of the wrist, reduce pressure on the carpal tunnel, and help restore the normal force structure of the patient's wrist.

Acupotomy have been used to treat CTS in China for many years. However, from the perspective of evidence-based medicine, the impact of needle cutters on CTS remains controversial. So far, a single systematic review of “ acupotomy ” and “carpal tube syndrome” has been published.^[17]^ The system, published in 2015, was designed to evaluate the therapeutic effects of 2 methods, comparing acupotomy treatment for CTS and acupotomy with concomitant steroid injection. Therefore, this systematic review hardly yielded any valuable conclusions. Three years have passed since the publication of the review in 2015, and many new experiments in this field have been published in the interim.^[18–22]^ However, an updated systematic review or research protocol on this issue has not yet been released, and it is not clear whether acupotomy treatment of CTS patients is effective and safe. Therefore, it is important to re-evaluate the system to obtain a relatively convincing conclusion as to whether acupotomy is a good choice for CTS patients.

## 2 Methods

### 2.1 Inclusion criteria for study selection

#### 2.1.1 Types of studies

The review will include randomized controlled trials (RCT) reported in any language for acupotomy therapy for CTS. Non-RCT and uncontrolled clinical trials will be excluded. Any study with a sample size of less than 10 will also be excluded from this review.

#### 2.1.2 Types of patients

Trials including participants who meet the CTS diagnostic criteria will be included.^[3,23,24]^ All eligible study participants are not subject to selection criteria based on age, gender, ethnicity, educational level, or economic status. Trials involving study participants who are not eligible for acupotomy treatment, such as fracture dislocation or space-occupying lesions and other serious illnesses, will be excluded.

#### 2.1.3 Types of interventions

The treatment group will be treated with acupotomy, and there is no limit to the needle material, treatment method, and treatment process.

Control interventions include acupuncture, placebo control, steroid injections, no treatment, Western medicine, routine care, and other conventional therapies. It will also include an evaluation of “Acupotomy-plus-another-treatment” compared to the same treatment alone. Studies that compare different needle insertions or different forms of acupotomy will be excluded.

#### 2.1.4 Types of outcome measures

##### 2.1.4.1 Primary outcomes

The primary outcome measures will be an improvement in the patient's symptoms and hand function. These are mainly evaluated by the Boston carpal tunnel questionnaire (BCTQ) and the visual analogue score (VAS).

##### 2.1.4.2 Secondary outcomes

The secondary outcome measures will include:

1. Electromyogram (EMG) changes (mainly changes in sensory nerve conduction velocity).
2. Safety: Measured by the incidence and severity of adverse reactions.
3. Acceptability of treatment as measured by withdrawal from the trial.

### 2.2 Search methods for the identification of studies

#### 2.2.1 Electronics searches

The following electronic databases will be searched cumulatively to October 2018: PubMed, EMBASE, Cochrane Library, MEDLINE, China National Knowledge Infrastructure (CNKI), China Biomedical Literature Database (CBM), Wanfang Database, and Chongqing VIP China Technology Journal Database (VIP). The acupotomy RCT of patients with carpal tunnel syndrome will be searched for in the database. The articles included will not be subject to language or publication status. The following medical search terms will be used: carpal tunnel syndrome, median nerve, median nerve crush, median nerve compression, delayed median nerve palsy, acupotomy, needle knife, small needle knife, randomized controlled trial, randomized controlled, randomized, controlled, and clinical trial. The Chinese translation of these search terms will be used in the Chinese databases. The PubMed search strategy is shown in Table 1.

**Table 1:**
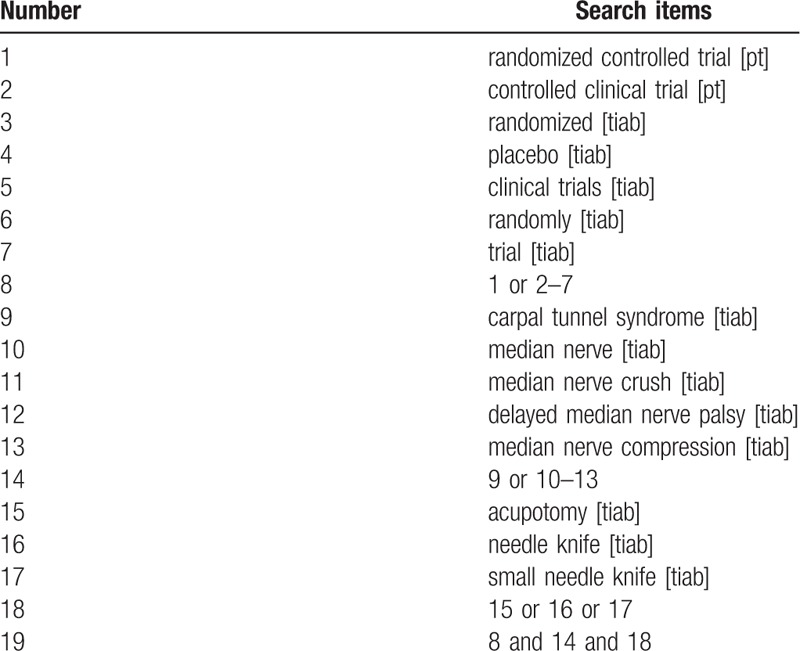
Search strategy used in PubMed database.

#### 2.2.2 Searching other resources

A reference list of studies and systematic reviews related to carpal tunnel syndrome and acupotomy will be examined to further determine other trials. We will also manually search for relevant conference papers and search for new trials related to the topic in Clinical Trials.gov and the WHO International Clinical Trial Registration Platform (ICTRP).

### 2.3 Data collection and analysis

#### 2.3.1 Selection of studies

**Figure 1:**
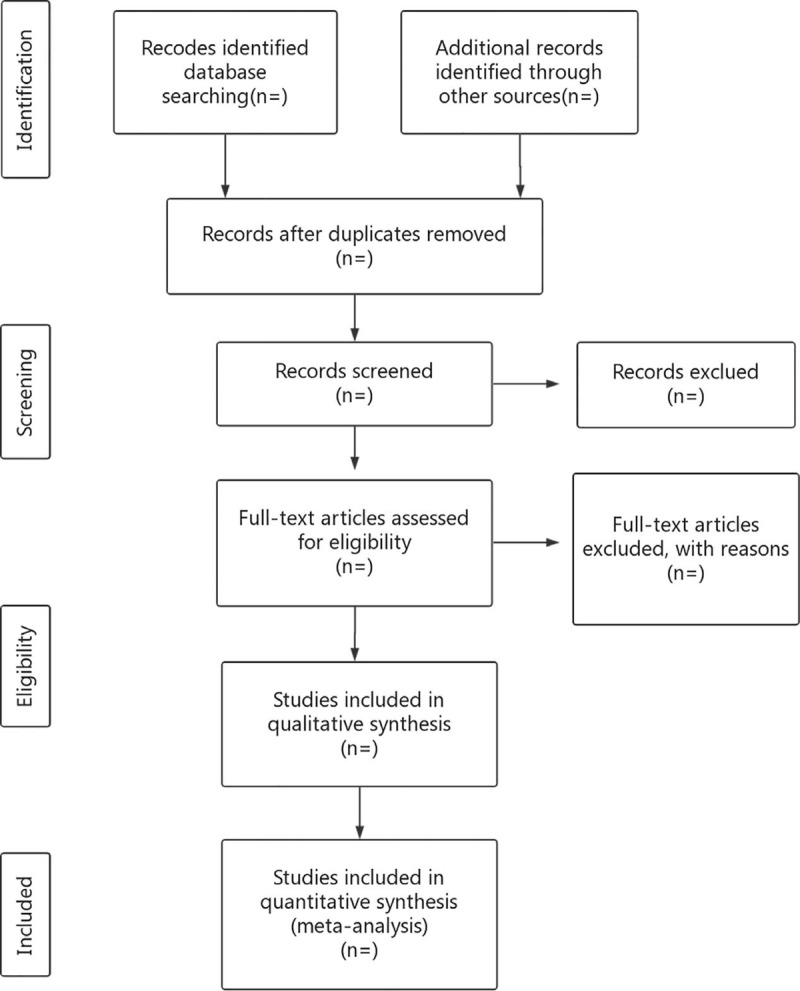
Flow diagram of the study selection process. CTS = carpal tunnel syndrome; RCT = randomized controlled trial.

Two researchers will import the retrieved documents into EndnoteX7 and eliminate duplicate data. Two independent reviewers will identify the titles and abstracts of all search studies based on inclusion criteria. Articles that are significantly sub-standard will be initially excluded by reviewing the title and abstract. The included studies will then be further evaluated by reading the full-text versions. Excluded studies will be listed in the table with the reasons for their exclusion. The results of the selection will be cross-checked by 2 reviewers. Any differences will be resolved through discussions with the third reviewer. The study selection procedure is shown in Figure 1.

#### 2.3.2 Data extraction and management

All research data will be extracted by 2 independent reviewers into a predefined data collection form. Any data discrepancies found during the cross-check will be resolved through discussion and recommendations from the third reviewer. This data collection form will include author information, publication time, participants, randomization, needle-knife interventions, control interventions, indicators, findings, and adverse events. If necessary, we will contact the trial author for further information.

#### 2.3.3 Assessment of risk of bias in included studies

Two independent reviewers will use the tools of the Cochrane Collaboration to assess the risk of bias for each included trial.^[25]^ The following 7 aspects will be assessed: generation of random sequences, allocation concealment, blinding of implementers and participants, blinding of outcome assessment, incomplete outcome data, selective reporting, and other biases as necessary. The risk of bias will be classified as low risk, high risk, and unclear. If the study lacks information on the risk of relative bias assessment, the trial author will be contacted for further information. The results of the evaluation will be cross-checked and resolved through discussion and arbitration by the third reviewer.

#### 2.3.4 Measures of treatment effect

A 95% confidence interval (CI) risk ratio will be used to represent an estimate of the impact of the dichotomous results. For continuous data, the average difference of 95% CI will be used for analysis. When the same results are measured in a variety of ways, the standardized mean difference of 95% CI will be used to indicate the magnitude of the intervention effect.

#### 2.3.5 Dealing with missing data

If feasible, the researchers will contact the appropriate author to obtain missing data. If the missing data are not available, we will perform the analysis based on the available data. In addition, if possible, we will conduct a sensitivity analysis to assess how sensitive the results are to reasonable changes in the assumptions made. If necessary, the potential impact of missing data on the final outcome of the review will be discussed.

#### 2.3.6 Assessment of heterogeneity

The *χ*^2^ test will be used to assess statistical heterogeneity. The *I*^2^ test will be used to quantify the inconsistencies between the included studies. If the *I*^2^ value is less than 50%, the study will not be considered heterogeneous while a value greater than 50% would indicate significant heterogeneity.

#### 2.3.7 Assessment of reporting bias

When more than 10 trials are included in the study, the funnel plot will be used to detect potential reported biases. When the image is not clear, the STATA 11.0 software will be quantified using the EGRATER test.

#### 2.3.8 Data synthesis

Data synthesis will be performed using RevMan software (V.5.3). If no substantial statistical heterogeneity is detected in the results, the fixed effects model will be used to summarize the data. If substantial statistical heterogeneity exists, the source of heterogeneity should be further analyzed. After excluding the effects of significant clinical heterogeneity, a random effects model will be used to pool the data. If there is significant clinical heterogeneity, a subgroup or sensitivity analysis or only descriptive analysis can be performed.

#### 2.3.9 Subgroup analysis

If there is significant heterogeneity in the included trials, we will perform a subgroup analysis based on the severity of CTS and the type of control intervention.

#### 2.3.10 Sensitivity analysis

If possible, a sensitivity analysis will be performed to verify the robustness of the review conclusions. The impact of methodological quality, sample size, and missing data will be assessed. In addition, the analysis will be repeated after the exclusion of low methodological quality studies.

#### 2.3.11 Grading the quality of evidence

We will assess the quality of evidence for all evidence through the Grading of Recommendations Assessment Development and Evaluation approach (GRADE), and rating it as very low, low, medium, or high.^[26–28]^ The results of the outcomes will be summarized in the “Summary of Findings” table.

#### 2.3.12 Ethics and dissemination

This review does not involve personal information or damages patient rights and therefore does not require ethical approval. The results of this systematic review will be published through peer-reviewed journals or conference reports. Due to the lack of relevant publications in this field, this review article will provide more evidence for acupotomy therapy for carpal tunnel syndrome by incorporating more recent studies into the analysis, leading to informed clinical practice and acupuncture research.

## 3 Discussion

The purpose of this study is to evaluate the efficacy and safety of acupotomy in the treatment of patients with carpal tunnel syndrome. The conclusions drawn from this review may be beneficial to CTS patients, clinicians, and decision makers. There are some potential limitations to this review. First, there may be a risk of heterogeneity in the severity of carpal tunnel syndrome and different types of acupotomy. Second, the reliability of the results depends to a large extent on the comprehensiveness and methodological quality of the main studies included in this review.

## Author contributions

**Data curation:** Qiaoyin Zhou, Yifeng Shen.

**Formal analysis:** Qiaoyin Zhou, Yan Jia, Zuyun Qiu.

**Investigation:** J.Sun, Yifeng Shen.

**Methodology:** Qiaoyin Zhou, J.Sun.

**Project administration:** Shiliang Li.

**Software:** Yan Jia, Zuyun Qiu.

**Supervision:** Shiliang Li.

**Validation:** Shiliang Li.

**Visualization:** Yifeng Shen.

**Writing – original draft:** Qiaoyin Zhou, Yifeng Shen.

**Writing – review & editing:** Shiliang Li.

## Supplementary Material

Supplemental Digital Content
