# Supplementary material for: Acupotomy for patients with carpal tunnel syndrome: A systematic review protocol
Source: Medicine (Baltimore). 2019 Dec 20;98(51):e18336. doi: 10.1097/MD.0000000000018336 (PMC6940147; doi:10.1097/MD.0000000000018336)
Supplement: Supplemental Digital Content [file medi-98-e18336-s001.pdf]

Systematic review

1. * Review title.

Give the working title of the review, for example the one used for obtaining funding. Ideally the title should state succinctly the interventions or exposures being reviewed and the associated health or social problems. Where appropriate, the title should use the PI(E)COS structure to contain information on the Participants, Intervention (or Exposure) and Comparison groups, the Outcomes to be measured and Study designs to be included.

Acupotomy for carpal tunnel syndrome: a systematic review protocol

2. Original language title.

For reviews in languages other than English, this field should be used to enter the title in the language of the review. This will be displayed together with the English language title.

3. * Anticipated or actual start date.

Give the date when the systematic review commenced, or is expected to commence.

01/12/2018

4. * Anticipated completion date.

Give the date by which the review is expected to be completed.

01/09/2020

5. * Stage of review at time of this submission.

Indicate the stage of progress of the review by ticking the relevant Started and Completed boxes. Additional information may be added in the free text box provided.
Please note: Reviews that have progressed beyond the point of completing data extraction at the time of initial registration are not eligible for inclusion in PROSPERO. Should evidence of incorrect status and/or completion date being supplied at the time of submission come to light, the content of the PROSPERO record will be removed leaving only the title and named contact details and a statement that inaccuracies in the stage of the review date had been identified.
This field should be updated when any amendments are made to a published record and on completion and publication of the review. If this field was pre-populated from the initial screening questions then you are not able to edit it until the record is published.

The review has not yet started: Yes

Review stage	Started	Completed
Preliminary searches	No	No
Piloting of the study selection process	No	No
Formal screening of search results against eligibility criteria	No	No
Data extraction	No	No
Risk of bias (quality) assessment	No	No
Data analysis	No	No

Provide any other relevant information about the stage of the review here (e.g. Funded proposal, protocol not yet finalised).

6. * Named contact.

The named contact acts as the guarantor for the accuracy of the information presented in the register record.

Yi-feng Shen

Email salutation (e.g. "Dr Smith" or "Joanne") for correspondence:

Mr Shen

7. * Named contact email.

Give the electronic mail address of the named contact.

syf721@qq.com

8. Named contact address

Give the full postal address for the named contact.

Department of Acupuncture and Moxibustion, China-Japan Friendship Hospital, Beijing

9. Named contact phone number.

Give the telephone number for the named contact, including international dialling code.

008618500235405

10. * Organisational affiliation of the review.

Full title of the organisational affiliations for this review and website address if available. This field may be completed as 'None' if the review is not affiliated to any organisation.

China-Japan Friendship Hospital

Organisation web address:

11. * Review team members and their organisational affiliations.

Give the title, first name, last name and the organisational affiliations of each member of the review team.

Affiliation refers to groups or organisations to which review team members belong.

Mr Yi-feng Shen. China-Japan Friendship Hospital
Dr Qiaoyin Zhou. College of Traditional Chinese Medicine, Fujian University of Traditional Chinese Medicine, Fuzhou, Fujian, China
Xiaojie Sun. Department of acupuncture-moxibustion, China-Japan Friendship hospital
Zuyun Qiu. Department of acupuncture-moxibustion, China-Japan Friendship hospital
Yan Jia. Department of acupuncture-moxibustion, China-Japan Friendship hospital
Professor Shiliang Li. Department of acupuncture-moxibustion, China-Japan Friendship hospital

12. * Funding sources/sponsors.

Give details of the individuals, organizations, groups or other legal entities who take responsibility for initiating, managing, sponsoring and/or financing the review. Include any unique identification numbers assigned to the review by the individuals or bodies listed.

China-Japan Friendship Hospital 2010 Research Fund-funded projects, 2010-MS-38, acupuncture treatment of cervical headache (cutaneous nerve entrapment) clinical research, 2011/10/-2013/10, 40, 000 yuan

13. * Conflicts of interest.

List any conditions that could lead to actual or perceived undue influence on judgements concerning the main topic investigated in the review.

None

14. Collaborators.

Give the name and affiliation of any individuals or organisations who are working on the review but who are not listed as review team members.

15. * Review question.

State the question(s) to be addressed by the review, clearly and precisely. Review questions may be specific or broad. It may be appropriate to break very broad questions down into a series of related more specific questions. Questions may be framed or refined using PI(E)COS where relevant.

To evaluate whether acupotomy is effective in treating carpal tunnel syndrome.

16. * Searches.

State the sources that will be searched. Give the search dates, and any restrictions (e.g. language or publication period). Do NOT enter the full search strategy (it may be provided as a link or attachment.)

Seven databases will be searched from their inception dates to December 2018, as follows: the English language databases PubMed, Embase, and The Cochrane Library, and the Chinese literature databases the Chinese Biomedical Literature Database [CBM], China National Knowledge Infrastructure [CNKI], and Wanfang.

Search strategy:

#1 (((carpal tunnel syndrome) OR CTS) OR Carpal Tunnel) OR Guyon syndrome

#2 ((acupotomy) OR small needle-knife) OR needle knife

#3 (((random[Text Word] OR randomized[Text Word]) OR control[TextWord]) OR controlment[Text Word]) OR trial[Text Word] AND "humans"[MeSH Terms]

#1AND#2AND#3

17. URL to search strategy.

Give a link to a published pdf/word document detailing either the search strategy or an example of a search strategy for a specific database if available (including the keywords that will be used in the search strategies), or upload your search strategy.Do NOT provide links to your search results.

Alternatively, upload your search strategy to CRD in pdf format. Please note that by doing so you are consenting to the file being made publicly accessible.

Do not make this file publicly available until the review is complete

18. * Condition or domain being studied.

Give a short description of the disease, condition or healthcare domain being studied. This could include health and wellbeing outcomes.

Carpal tunnel syndrome (CTS) is a condition best described as a 'constellation of symptoms' more complex than most. A CTS patient may therefore present an 'ill structured clinical problem', the diagnosis of which must be approached with caution, and with a thorough understanding of the contributory pathophysiological mechanisms.

19. * Participants/population.

Give summary criteria for the participants or populations being studied by the review. The preferred format includes details of both inclusion and exclusion criteria.

Patients of any gender, age, race or nationality suffering from carpal tunnel syndrome.

20. * Intervention(s), exposure(s).

Give full and clear descriptions or definitions of the nature of the interventions or the exposures to be reviewed.

The treatment group will receive acupotomy (no limits will be imposed on the needle materials, treatment methods, or on the course of treatment).

21. * Comparator(s)/control.

Where relevant, give details of the alternatives against which the main subject/topic of the review will be compared (e.g. another intervention or a non-exposed control group). The preferred format includes details of both inclusion and exclusion criteria.

The control group will receive an internationally recognized therapy such as block therapy or no treatment, and acupuncture will also be included.

Acupotomy with adjuvant therapy versus the same therapy alone will also be investigated.

Studies comparing two different types of acupotomy or surgical procedures will be excluded.

22. * Types of study to be included.

Give details of the types of study (study designs) eligible for inclusion in the review. If there are no restrictions on the types of study design eligible for inclusion, or certain study types are excluded, this should be stated. The preferred format includes details of both inclusion and exclusion criteria.

We will include published randomized controlled clinical trials (RCTs) and quasi- randomized controlled clinical trials (quasi-RCTs), comparing any form of acupotomy with/without additional treatment against placebo or sham or no treatment or same additional treatment.

23. Context.

Give summary details of the setting and other relevant characteristics which help define the inclusion or exclusion criteria.

24. * Main outcome(s).

Give the pre-specified main (most important) outcomes of the review, including details of how the outcome is defined and measured and when these measurement are made, if these are part of the review inclusion criteria.

Pain.

Timing and effect measures

Visual analog scale (VAS)-based measurements.

25. * Additional outcome(s).

List the pre-specified additional outcomes of the review, with a similar level of detail to that required for main outcomes. Where there are no additional outcomes please state ‘None’ or ‘Not applicable’ as appropriate to the review

Treatment efficiency.

Timing and effect measures

The percentage of patients with improved symptoms.

26. * Data extraction (selection and coding).

Describe how studies will be selected for inclusion. State what data will be extracted or obtained. State how this will be done and recorded.

All research data will be extracted by two independent reviewers into a predefined data collection form. Any data discrepancies found during the cross-check will be resolved through discussion and recommendations from the third reviewer. This data collection form will include author information, publication time, participants, randomization, needle-knife interventions, control interventions, indicators, findings, and adverse events. If necessary, we will contact the trial author for further information.

27. * Risk of bias (quality) assessment.

Describe the method of assessing risk of bias or quality assessment. State which characteristics of the studies will be assessed and any formal risk of bias tools that will be used.

We will apply Cochrane systematic review methods, and the Cochrane risk of bias tool, as recommended by the Cochrane Reviewer’s Handbook 5.0.24, will be used to assess the quality of the ultimate included studies. The specific items evaluated are follows: random sequence generation, allocation concealment, blinding, completeness of outcome data, selective outcome reporting, and other bias. The risk of bias for

each item will be graded as 'low risk of bias', 'unclear risk of bias' or 'high risk of bias'. Authors' judgments about each risk of bias item will be presented as percentages across all included studies.

If we cannot acquire the information we need to assess the articles, we will contact the corresponding author by telephone to get establish clarity and request any missing information. Two of the authors will conduct these activities independently, and any disagreements will be resolved by an arbiter.

28. * Strategy for data synthesis.

Provide details of the planned synthesis including a rationale for the methods selected. This **must not be generic text** but should be **specific to your review** and describe how the proposed analysis will be applied to your data.

Comparisons will be made between any form of acupotomy and placebo or sham or no treatment with/without same additional treatment is given to both groups. The data of the study we will include may be divided into two cases, depending on whether the data are suitable for meta-analysis. If the meta-analysis is not adaptive because of the heterogeneity of different participants, interventions, comparisons, outcomes et al, we will build a set of forms and summaries to conduct a qualitative description. If the meta-analysis is suitable for the data we will collect because of their good performance in homogeneity, we will apply the meta-analysis to conduct a quantitative analysis. We plan to use the Review Manager Software, RevMan 5.0.24. For the data is going to be done with the meta-analysis, we should do the tests for heterogeneity first. We plan to test heterogeneity by using a standard ?² test. When there are sufficient similar studies of relatively high heterogeneity (P0.10, I^2 =50%), we will consider applying the fixed-effect model to do the data synthesis. For dichotomous data, we will take relative risk (RR) and take mean difference (MD) for continuous data; both of them will be with 95% confidence intervals (CI). While if the studies are of relatively low heterogeneity (P0.10), we would do the subgroup analysis or the sensitivity analysis. Finally, we will test the overall effect by using the Z score with the significance set at P=0.05. On the condition of including more than 10 trials, funnel plots will be applied to detect whether there is publication bias or not.

If there is found to be significant heterogeneity in the included trials, we will conduct subgroup analyses, as described below.

29. * Analysis of subgroups or subsets.

State any planned investigation of 'subgroups'. Be clear and specific about which type of study or participant will be included in each group or covariate investigated. State the planned analytic approach.

Subgroup analysis will be carried out, if appropriate, based on the severity of the carpal tunnel syndrome, and on the types of acupotomy used.

30. * Type and method of review.

Select the type of review and the review method from the lists below. Select the health area(s) of interest for your review.

Type of review

Cost effectiveness
No

Diagnostic

No

Epidemiologic
No

Individual patient data (IPD) meta-analysis
No

Intervention
Yes

Meta-analysis
Yes

Methodology
No

Narrative synthesis
No

Network meta-analysis
No

Pre-clinical
No

Prevention
No

Prognostic
No

Prospective meta-analysis (PMA)
No

Review of reviews
No

Service delivery
No

Synthesis of qualitative studies
No

Systematic review
Yes

Other
No

Health area of the review

Alcohol/substance misuse/abuse
No

Blood and immune system
No

Cancer
No

Cardiovascular
No

Care of the elderly
No

Child health
No

Complementary therapies
Yes

Crime and justice

No

Dental
No

Digestive system
No

Ear, nose and throat
No

Education
No

Endocrine and metabolic disorders
No

Eye disorders
No

General interest
No

Genetics
No

Health inequalities/health equity
No

Infections and infestations
No

International development
No

Mental health and behavioural conditions
No

Musculoskeletal
Yes

Neurological
No

Nursing
No

Obstetrics and gynaecology
No

Oral health
No

Palliative care
No

Perioperative care
No

Physiotherapy
No

Pregnancy and childbirth
No

Public health (including social determinants of health)
No

Rehabilitation
No

Respiratory disorders
No

Service delivery
No

Skin disorders
No

Social care
No

Surgery
No

Tropical Medicine
No

Urological
No

Wounds, injuries and accidents
No

Violence and abuse
No

31. Language.

Select each language individually to add it to the list below, use the bin icon to remove any added in error.
Chinese-Simplified
English

There is not an English language summary

32. * Country.

Select the country in which the review is being carried out from the drop down list. For multi-national collaborations select all the countries involved.

China

33. Other registration details.

Give the name of any organisation where the systematic review title or protocol is registered (such as with The Campbell Collaboration, or The Joanna Briggs Institute) together with any unique identification number assigned. (N.B. Registration details for Cochrane protocols will be automatically entered). If extracted data will be stored and made available through a repository such as the Systematic Review Data Repository (SRDR), details and a link should be included here. If none, leave blank.

34. Reference and/or URL for published protocol.

Give the citation and link for the published protocol, if there is one

Give the link to the published protocol.

Alternatively, upload your published protocol to CRD in pdf format. Please note that by doing so you are consenting to the file being made publicly accessible.

No I do not make this file publicly available until the review is complete

Please note that the information required in the PROSPERO registration form must be completed in full even if access to a protocol is given.

35. Dissemination plans.

Give brief details of plans for communicating essential messages from the review to the appropriate audiences.

Do you intend to publish the review on completion?

No

36. Keywords.

Give words or phrases that best describe the review. Separate keywords with a semicolon or new line. Keywords will help users find the review in the Register (the words do not appear in the public record but are included in searches). Be as specific and precise as possible. Avoid acronyms and abbreviations unless these are in wide use.

37. Details of any existing review of the same topic by the same authors.

Give details of earlier versions of the systematic review if an update of an existing review is being registered, including full bibliographic reference if possible.

38. * Current review status.

Review status should be updated when the review is completed and when it is published. For newregistrations the review must be Ongoing.
Please provide anticipated publication date

Review_Ongoing

39. Any additional information.

Provide any other information the review team feel is relevant to the registration of the review.

40. Details of final report/publication(s).

This field should be left empty until details of the completed review are available.

Give the link to the published review.
